# Treatment of Placenta Previa

**Published:** 1897-04

**Authors:** 


### TREATMENT OF PLACENTA PREVIA.

Baumm (*Centralbl f. Gynakol.*, No. 39, 1896,) recommends external version in placenta previa, that the presentation being converted into a pelvic one, the hemorrhage may be arrested by drawing down and keeping up traction on a foot. The version is generally possible, as the placenta prevents the early engagement of the head; after it has been performed, if the os is not sufficiently dilated to admit two fingers, one must, when bleeding begins, apply a tampon and wait; if the genitals are relaxed, it is generally easy, even without an anesthetic, to bring down a foot, and by moderate and steady traction, to deliver the woman without further loss of blood. If the bleeding be severe and alarming, it is better to employ combined podalic version at once, or to apply a tampon before attempting external version.—*British Medical Journal.*

---

PROFESSOR PAJOT, the distinguished French obstetrician, died recently in Paris, at the age of eighty. He was the founder and director of the *Annales de Gynecologie*, and the founder and first President of the Societe d'Obstrique et de Gynecologie de Paris. Pajot was a man of great acuteness and originality of mind, and as a practical obstetrician he was almost without a rival in France. He was noted also for his caustic wit, which made him many enemies. Few of his epigrams were as harmless as his epitaph on Civiale, the famous lithotritist, which has been thus translated:

"For Civiale, who's dead and gone,
Though sympathetic tears may gush,
Set not upon his grave a stone
Which he would surely rise and crush."

---

The *Laryngoscope*, published in St. Louis, has been selected as the official organ, for the year 1897, of the Laryngological Section of the New York Academy of Medicine. This selection and the great probability of the same journal being chosen by other Laryngological, Rhinological and Otological Societies as their official organ, would indicate that *The Laryngoscope* has become what its proprietors stated they intended to make it, *i. e.*, The American Journal of Record for the specilaties represented.

---

EVERY medical man should be a member of a medical society. He will never know how great a man he is till some one praises him in a discussion, nor how small a man till some pompous fellow-member takes him to task; but all these frictions serve but to round and smooth a busy life, and no one can do without it who desires to be a physician in the highest acceptancy and not a man who doctors.—*Atlantic Medical Weekly.*

